# Genetic and epigenetic characterization of posterior pituitary tumors

**DOI:** 10.1007/s00401-021-02377-1

**Published:** 2021-10-18

**Authors:** Simone Schmid, David A. Solomon, Eilis Perez, Anne Thieme, Bette K. Kleinschmidt-DeMasters, Caterina Giannini, Annekathrin Reinhardt, Sylvia L. Asa, Ozgur Mete, Damian Stichel, Christin Siewert, Carsten Dittmayer, Martin Hasselblatt, Werner Paulus, Christoph Nagel, Patrick N. Harter, Jens Schittenhelm, Jürgen Honegger, Elisabeth Rushing, Roland Coras, Stefan M. Pfister, Rolf Buslei, Arend Koch, Arie Perry, David T. W. Jones, Andreas von Deimling, David Capper, M. Beatriz Lopes

**Affiliations:** 1 Charité – Universitätsmedizin Berlin, corporate member of Freie Universität Berlin and Humboldt-Universität zu Berlin, Department of Neuropathology, Charitéplatz 1, Berlin, Germany; 2 Department of Pathology, University of California, San Francisco, CA USA; 3 German Cancer Consortium (DKTK), Partner Site Berlin, German Cancer Research Center (DKFZ), Heidelberg, Germany; 4 Department of Pathology, University of Colorado, Aurora, CO USA; 5 Department of Pathology and Laboratory Medicine, Mayo Clinic, Rochester, MN USA; 6 Department of Neuropathology, Institute of Pathology, Heidelberg University Hospital, Heidelberg, Germany; 7 Clinical Cooperation Unit Neuropathology, German Cancer Consortium (DKTK), German Cancer Research Center (DKFZ), Heidelberg, Germany; 8 Department of Pathology, Case Western Reserve University, Cleveland, OH USA; 9 Department of Pathology, University Health Network, Toronto, ON Canada; 10 Institute of Neuropathology, University Hospital Münster, Münster, Germany; 11 Division of Neurosurgery, Sankt Gertrauden Hospital, Berlin, Germany; 12 Institute of Neurology (Edinger Institute), Goethe University, Frankfurt, Germany; 13 German Cancer Consortium (DKTK), Partner Site Frankfurt/Mainz, Frankfurt am Main, Germany; 14 German Cancer Research Center (DKFZ), Heidelberg, Germany; 15 Frankfurt Cancer Institute (FCI), Frankfurt am Main, Germany; 16 Center for Neuro-Oncology, Comprehensive Cancer Center Tübingen-Stuttgart, University Hospital Tübingen, Eberhard Karls University, Tübingen, Germany; 17 Department of Neuropathology, University Hospital Tübingen, Eberhard Karls University, Tübingen, Germany; 18 Department of Neurosurgery, University of Tübingen, Tübingen, Germany; 19 Institute of Neuropathology, University Hospital and University of Zurich, Zurich, Switzerland; 20 Department of Neuropathology, Institute of Neuropathology, Affiliated Partner of the ERN EpiCARE, Universitätsklinikum Erlangen, Friedrich-Alexander-University of Erlangen-Nürnberg (FAU), Schwabachanlage 6, 91054 Erlangen, Germany; 21 Hopp Children’s Cancer Center (KiTZ), Heidelberg, Germany; 22 Department of Pediatric Oncology, Hematology and Immunology, University Hospital Heidelberg, Heidelberg, Germany; 23 Division of Pediatric Neurooncology, German Cancer Consortium (DKTK), German Cancer Research Center (DKFZ), Heidelberg, Germany; 24 Institute of Pathology, Sozialstiftung Bamberg, Buger Str. 80, 96049 Bamberg, Germany; 25 Pediatric Glioma Research Group, German Consortium for Translational Cancer Research (DKTK), German Cancer Research Center (DKFZ), Heidelberg, Germany; 26 Department of Pathology, Neuropathology, University of Virginia Health System, Charlottesville, Virginia USA

**Keywords:** Pituicytoma, Spindle cell oncocytoma, Granular cell tumor, Posterior pituitary gland neoplasms, Molecular neuropathology, Brain tumor

## Abstract

Pituicytoma (PITUI), granular cell tumor (GCT), and spindle cell oncocytoma (SCO) are rare tumors of the posterior pituitary. Histologically, they may be challenging to distinguish and have been proposed to represent a histological spectrum of a single entity. We performed targeted next-generation sequencing, DNA methylation profiling, and copy number analysis on 47 tumors (14 PITUI; 12 GCT; 21 SCO) to investigate molecular features and explore possibilities of clinically meaningful tumor subclassification. We detected two main epigenomic subgroups by unsupervised clustering of DNA methylation data, though the overall methylation differences were subtle. The largest group (*n* = 23) contained most PITUIs and a subset of SCOs and was enriched for pathogenic mutations within genes in the MAPK/PI3K pathways (12/17 [71%] of sequenced tumors: *FGFR1* (3), *HRAS* (3), *BRAF* (2), *NF1* (2), *CBL* (1), *MAP2K2* (1), *PTEN* (1)) and two with accompanying *TERT* promoter mutation. The second group (*n* = 16) contained most GCTs and a subset of SCOs, all of which mostly lacked identifiable genetic drivers. Outcome analysis demonstrated that the presence of chromosomal imbalances was significantly associated with reduced progression-free survival especially within the combined PITUI and SCO group (*p* = 0.031). In summary, we observed only subtle DNA methylation differences between posterior pituitary tumors, indicating that these tumors may be best classified as subtypes of a single entity. Nevertheless, our data indicate differences in mutation patterns and clinical outcome. For a clinically meaningful subclassification, we propose a combined histo-molecular approach into three subtypes: one subtype is defined by granular cell histology, scarcity of identifiable oncogenic mutations, and favorable outcome. The other two subtypes have either SCO or PITUI histology but are segregated by chromosomal copy number profile into a favorable group (no copy number changes) and a less favorable group (copy number imbalances present). Both of the latter groups have recurrent MAPK/PI3K genetic alterations that represent potential therapeutic targets.

**Supplementary Information:**

The online version contains supplementary material available at 10.1007/s00401-021-02377-1.

## Introduction

Spindle cell oncocytoma, pituicytoma, and granular cell tumor of the sellar region are rare pituicyte-derived neoplasms. They are listed as independent entities in the 2016 WHO Classification of Tumours of the Central Nervous System and are assigned as WHO grade I neoplasms [37] due to their typically low-grade histological appearance, slow growth, and benign clinical course. Epidemiological data are not available for these rare neoplasms. A meta-analysis of literature published in either English or Spanish language reported around 270 examples [19].

Granular cell tumors are typically composed of cells with isomorphic, round to polygonal cell bodies, rather small and often eccentric nuclei, and abundant granular cytoplasm. Pituicytomas most closely resemble the majority of normal pituicytes in the posterior pituitary, which are spindled and elongated cells arranged in fascicles. The morphology of spindle cell oncocytomas is less clearly defined. These tumors vary in appearance and may show, among other patterns, spindled and/or epithelioid cells, with varying oncocytic changes and fascicular growth [37, 38].

All three tumors originate in a very specific and confined region of the CNS, the posterior lobe, and the infundibulum of the pituitary gland, also called the neurohypophysis, which is formed during embryonic development from a protrusion of the developing diencephalon. In early gestation, it co-localizes with the early adenohypophysis, which derives from the oral ectoderm of Rathke’s pouch and together they form the pituitary gland in the sella turcica [4, 31, 44, 59].

Due to the variety of cell types found in this small anatomic space, there have been various theories about the cellular origin of spindle cell oncocytomas, pituicytomas, and granular cell tumors of the sella [9, 52, 54]. Currently, it is widely accepted that all three tumors derive from pituicytes, the supportive glial parenchymal cells of the posterior pituitary, which also show a spectrum of morphologies in normal conditions [57, 58]. This is substantiated by the nuclear expression of thyroid transcription factor-1 (TTF-1, encoded by the *NKX2-1* gene) which is a marker expressed in normal pituicytes, spindle cell oncocytomas, pituicytomas, and granular cell tumors of the sella [35, 36, 64]. It has therefore been proposed that these tumor types should be classified as subtypes of a single entity [20, 41]. Interestingly, subependymal giant cell astrocytomas (SEGA), chordoid gliomas of the third ventricle, and hypothalamic gangliocytomas or neurocytomas have also been shown to express TTF-1, and it has been proposed that TTF-1 positive tumors may represent a spectrum of early forebrain derived neoplasms [2, 21–23, 42, 64].

In 2018, it was shown that CNS tumors can be distinguished and reliably classified by genome-wide DNA methylation profiling [7]. In the course of the increased utilization of the DNA methylation-based classifier tool (https://www.molecularneuropathology.org/mnp), several new entities have been recognized that typically share group-specific DNA methylation patterns, often in combination with common molecular features, like common gene fusions, mutations, or other genomic variants [14, 46, 56, 61].The initial classifier cohort also included the profiles of 29 posterior pituitary lobe tumors, with representatives of each of the three histological types [7]. In that initial study, no separation of pituicytoma, granular cell tumor, and spindle cell oncocytoma was observed and they were therefore assigned to a single DNA methylation class in the brain tumor classifier reference set v11b4 [7].

In this present study, we performed integrated histological and molecular analysis of an extended cohort of a total of 47 posterior pituitary tumors. We here focus on the question of molecular differences between the three histologic types using DNA methylation analysis, copy number analysis, and targeted next-generation sequencing of approximately 500 cancer-associated genes [43, 60]. We further included clinical follow up data to investigate potential associations of histological or molecular features with disease outcome and to develop a meaningful subgrouping of posterior pituitary tumors based on histological and molecular traits.

## Materials and methods

### Tissue samples

Formalin-fixed, paraffin-embedded (FFPE) tumor tissue from 47 neoplasms of the posterior pituitary gland were collected from the pathology archives of neuropathology / pathology departments at the University Hospital Erlangen (*n* = 10), University of Virginia (*n* = 6), Mayo Clinic (*n* = 6), University Hospital Heidelberg and Mannheim (*n* = 6), University of Colorado (*n* = 4), University of California San Francisco (*n* = 4), University Hospital Charité Berlin (*n* = 3), University Hospital Münster (*n* = 2), University Hospital Frankfurt am Main (*n* = 2), University Network Hospital Toronto (*n* = 2), University Hospital Tübingen (*n* = 1), and University Hospital Zürich (*n* = 1). The studied tumors had been resected during the period of 1986–2018 and clinically diagnosed as either pituicytoma (*n* = 14), spindle cell oncocytoma (*n* = 21), or granular cell tumor of the sella (*n* = 12). All cases were collected from centers with extensive experience in pituitary tumor diagnostics, and were well-characterized by immunohistochemistry and/or electron microscopy. Supplementary Table 1 summarizes the external diagnostic data. The original institutional pathologic diagnosis was used for this study (i.e. we did not centrally reclassify tumors). Thirty-four cases were initial tumor resections without prior therapy, four cases were recurrent tumors without prior radiotherapy, four cases were recurrences after radiotherapy, and for five cases the treatment data was not fully retrievable. For three of the recurrent cases (one case after irradiation and two cases without irradiation), DNA methylation analyses was additionally performed on the primary tumor to investigate if the presence of copy number changes was stable over time. The DNA methylation data of twenty-nine tumors was previously published [7]. In addition, methylation data were newly generated and analyzed from non-neoplastic samples of infundibulum (*n* = 7) and posterior pituitary lobe (*n* = 6). These non-neoplastic tissue samples were from surgical specimens (*n* = 4) or post-mortem autopsy specimens (*n* = 9). Ethical approval was given by the local ethics committee of the Charité (EA2/151/18). For the supplying institutions, usage of material was approved by local Institutional Review Boards.

### Clinical data

Whenever possible the following patient data were provided by the supplying institution: patient sex, age at surgery, initial diagnosis, disease stage (primary lesion, first recurrence, second recurrence), prior therapies (e.g. radiation), and disease course (time to progression on MRI or time to second resection, disease associated death). Second resections represented tumor regrowth and thus true recurrences in all cases. Imaging could not be reviewed in a structured fashion because of the many participating institutions and the changes of imaging techniques within the acquisition period (1986–2018).

### Evaluation of histological features

H&E stained slides were available for evaluation of histological features from 46 of 47 tumors included in this study. For each case, two experienced pathologists (S.S. and D.C.) evaluated and graded a number of morphological features on H&E slides using a multihead microscope. Consensus was reached by discussion using the following scale: feature present in less than 1% of tumor cells (graded as “not present”), in 1–10% of tumor cells (“slight”), in 10–50% (“moderate”) of tumor cells, or in over 50% of tumor cells (“high”). The following features were graded in this way: fascicular growth of spindled cells, lobulated growth, granular cells (not differentiating between oncocytic and lysosomal granularity), rounded to polygonal cells with distinct cell borders (epithelioid appearance), nuclear pleomorphism, prominent nucleoli, necrosis, and intraparenchymal sclerosis. In addition, we evaluated cell density by counting cells in an area of 1 mm^2^. Lymphocytic infiltrates were assessed by counting blood vessels with lymphocytic cuffs (defined as more than 10 lymphocytes surrounding one vessel) in the whole tissue section and scored as: not present—lymphocytic cuffs around 0 to 1 vessel; low—cuffs around 2 to 5 vessels; moderate—cuffs around 6 to 10 vessels; high—cuffs around more than 10 vessels. Further, mitotic figures were counted in 10 high power fields (HPF) [1 high power field = 0.26 mm^2^] of tumor tissue. Mitotic activity was scored as: not present—0 mitotic figures; low—1 mitotic figure in 10 HPF; moderate—2 mitotic figures in 10 HPF; high—3 or more mitotic figures in HPF.

### Anti-mitochondrial antigen immunohistochemistry

Immunohistochemistry for mitochondrial antigen was performed using antibody clone 113-1 from BioGenex on whole formalin-fixed, paraffin-embedded tissue sections at 1:500 dilution following ER1 antigen retrieval for selected cases. Immunostaining was performed in a Leica BOND-III automated stainer with diaminobenzidine used as the chromogen, followed by hematoxylin counterstain.

### DNA methylation analysis

DNA methylation and copy number analyses were performed using the Infinium Methylation450k and EPIC BeadChip array platforms (Illumina, USA). All analyses were performed according to the manufacturer’s instructions. In brief, DNA was extracted from FFPE tumor samples using the Maxwell RSC FFPE Plus DNA Purification Kit (Promega, USA). After bisulfite conversion using the Zymo EZ Methylation Kit (Zymo Research Irvine, USA), the Infinium HD FFPE DNA Restore Kit was used for DNA restoration. The beadchips were scanned on the iScan system (Illumina, USA). The unprocessed output data (.idat files) from the iScan reader were checked for general quality measures as indicated by the manufacturer. DNA methylation based classification was performed for both 450 k and 850 k data using the publically available “brain tumor classifier”, version v11b4 (https://www.molecularneuropathology.org/mnp) [7].

### Dimensionality reduction

For DNA methylation data, dimensionality reduction was performed. All analyses were conducted on R version 3.6.1 (2019–07-05) – "Action of the Toes" [49]. Raw summary intensity signals were processed using the R package minfi, version 1.26.2. [1]. rgSets of EPIC array and 450k array data from our tumor set and from the brain tumor classifier reference data set [7] were merged. A combined rgSet in 450k output format was generated using the set of overlap probes on both array types. Functional normalization preprocessing was done for all samples [15]. Quality control and filtering of probes were performed as recommended [40]. The m + u method implemented in the detectP function of the minfi package compares the total DNA signal (methylated + unmethylated) for each position to the background signal level. The background is estimated using negative control positions, assuming a normal distribution. Only samples with a mean *p* value of < 0.01 and only probes with a *p* value less than 0.01 in over 90% of the samples were kept for further analysis. Probes on sex chromosomes, probes of CpG sites that include single-nucleotide polymorphisms (SNP), as well as cross reactive probes were excluded [12]. After filtering and normalization, 415,907 probes remained for further analysis. The variance in the data set was estimated by applying standard deviation over the β-values of all probes of all samples. The 25,000 probes showing the highest variance were kept for dimensionality reduction using the Rtsne package, version: 0.13 [33, 34, 39]. A Pearson distance matrix was used as input object, theta of 0.5, perplexity of 10 and iteration of 1000 were used. Basic plots were generated with the ggplot2 package version: 3.2.1 [62]. Graphs were finalized with Adobe Illustrator CC 2017, version 2017.0.2 (1998–2017 Adobe). The unprocessed.idat files are available from the NCBI Gene Expression Omnibus (https://www.ncbi.nlm.nih.gov/geo/) under study ID GSE185041.

### Unsupervised hierarchical cluster analysis

We performed unsupervised hierarchical clustering analysis for further investigation of variation in our DNA methylation data and to determine the optimal number of clusters. These analyses were done using the R packages base:stats, NbClust (v.3.0) and ComplexHeatmap (v.2.0.0) [11, 18]. β-values of the 25,000 most variant CpG probes across all samples were used for analysis. We found Pearson distance measure and complete linkage clustering method yielded optimum results in our dataset, and these were used for further analysis. Further characterization of possible outlier tumors was done using the R function estimateCellCounts() implemented in the R package minfi and with the RFpurity package [28].

### Differentially methylated regions and gene set analysis

Analysis of differentially methylated regions (DMRs) was done using the R packages limma (v.3.46.0) and DMRcate (v.2.4.1) as previously described [40, 47, 51]. Cases from the reference set of the brain tumor classifier (pilocytic astrocytoma, posterior fossa; pilocytic astrocytoma, midline; and pleomorphic xanthoastrocytoma) were included in the analysis [7]. Normalization and filtering of probes were the same as stated above and followed established workflow recommendations [40]. Investigation of DMRs was performed with a cutoff of false discovery rate (FDR) > 0.05 and a mean beta value difference of ≥ 0.2 across the CpGs.

Gene set enrichment analysis was performed on the CpG site level comparisons using the R package methylGSA (v.1.8.0) [50]. The function methylRRA() was used to adjust for number of CpGs per each gene. Further annotation of the DMR results was done using the R package clusterProfiler (v3.18.0) and the public databases Ensembl, Reactome and The Gene Ontology (GO) knowledgebase [3, 16, 26, 27, 63].

### Targeted next-generation DNA sequencing analysis

A total of 37 of 47 tumor samples had sufficient material available for targeted DNA sequencing analysis. Capture-based next-generation sequencing was performed using an assay that targets all coding exons of 479 cancer-related genes, select introns and upstream regulatory regions of 47 genes to enable detection of structural variants including gene fusions, and DNA segments at regular intervals along each chromosome to enable genome-wide copy number and zygosity analysis, with a total sequencing footprint of 2.8 Mb (UCSF500 Cancer Panel; Supplementary Table 2) [32]. Multiplex library preparation was performed using the KAPA Hyper Prep Kit (Roche) according to the manufacturer’s specifications using 250 ng of sample DNA. Hybrid capture of pooled libraries was performed using a custom oligonucleotide library (Nimblegen SeqCap EZ Choice). Captured libraries were sequenced as paired-end 100 bp reads on an Illumina HiSeq 2500 instrument. Sequence reads were mapped to the reference human genome build GRCh37 (hg19) using the Burrows-Wheeler aligner (BWA). Recalibration and deduplication of reads was performed using the Genome Analysis Toolkit (GATK). Single nucleotide variant and insertion/deletion mutation calling was performed with Unified Genotyper, FreeBayes, and PinDel. Structural variant calling was performed with Delly. Variant annotation was performed with Annovar. Single nucleotide variants, insertions/deletions, and structural variants were visualized and verified using Integrative Genomics Viewer. Genome-wide copy number analysis based on on-target and off-target reads was performed by CNVkit and visualized using Nexus Copy Number (Biodiscovery).

All single nucleotide variants and insertions/deletions that were present at less than 0.1% frequency in each of the ExAC, ESP6500, or 1000 genomes human population datasets were filtered for further evaluation and predicted pathogenicity. Each of these filtered variants were reviewed against known cancer genomics data in the COSMIC (http://cancer.sanger.ac.uk/cosmic) and cBioPortal (http://www.cbioportal.org/) databases and also assessed where in the protein the mutations are located (i.e. within the active site of the kinase domain), the predicted effect of the variant, the mutant allele frequency, and other variables.

### Analysis of copy number variation

Changes of chromosomal copy number were determined from both methylation array data and the targeted next-generation sequencing data. Calculation of copy number profiles from methylation array data were done using the conumee package, version: 1.18.0 [25]. The evaluation was carried out manually with consideration of the histological tumor cell content for the evaluation of chromosomal gains or losses. In general, changes were considered potentially relevant if the intensity ratio of a segment deviated from the baseline by more than 0.1 [8]. In addition, we created summary copy number profiles for various groups. This analysis was done using an adaption of the conumee script (provided by Dr. Damian Stichel, Neuropathology Heidelberg).

### Progression free survival analysis

Outcome data were available for 37 patients. Progression-free survival analysis was performed using the R packages survival, package version: 2.44-1.1 and survminer, package version: 0.4.6. [30]. We used Kaplan–Meier estimates to investigate differences in progression free survival. Availability of clinical data was limited for a subset of patients. The start point for outcome analysis was the date of the first histological diagnosis of a posterior pituitary tumor. As an “event” for progression analysis, we defined either the performance of a further surgical intervention (using the date of surgery as event date) or if available tumor progression on MRI (using the date of the MRI as event date). Seven patients died during follow up period (four died of unknown reasons, three died of post-surgery complications). Definite tumor-associated death could not be determined for the patients in this series. Median follow up time was 5.1 years (range 11 days to 17 years).

## Results

### Evaluation of histological features

**Fig. 1:**
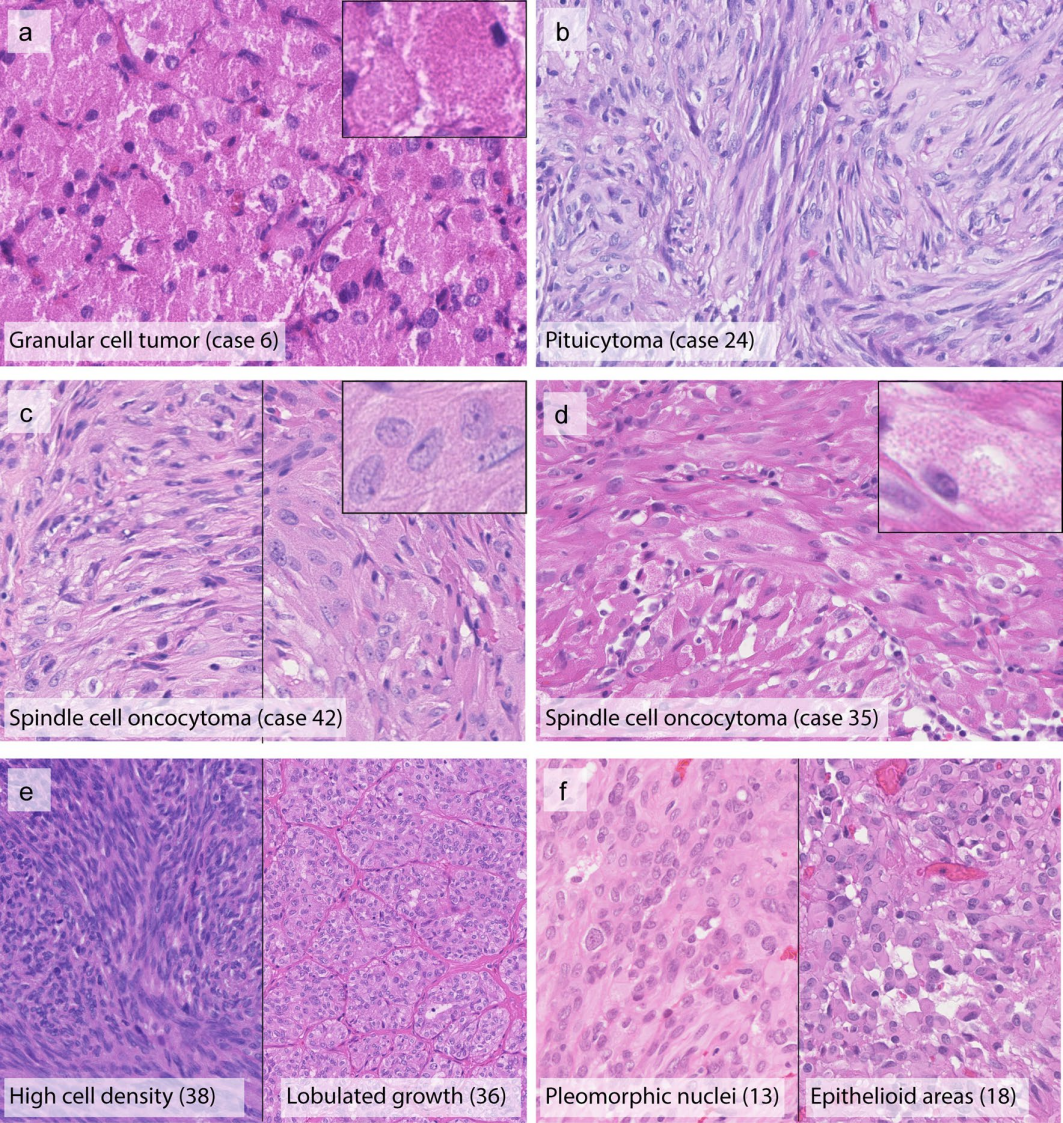
Representative hematoxylin and eosin stained images illustrating the histomorphological spectrum of the different posterior pituitary tumor entities. Granular cell tumors typically present with polygonal cells, granular cytoplasm and small eccentric nuclei (**a**). Pituicytomas typically present with predominantly spindled cells lacking granular or oncocytic changes (**b**). Spindle cell oncocytomas have more variable histological appearances; prototypical specimens show storiform or fascicular growth (**c**). Some degree of granular cytoplasmic changes was observed among spindle cell oncocytomas of this series. Some samples showed only focal granular cytoplasm (**c**) whereas those changes were prominent in other tumors (**d**). Some tumors presented with morphological features such as high cell density, lobulated growth pattern (**e**), pleomorphic nuclei, or epithelioid cytology (**f**). Original magnification of all images 400×, inset 800×

**Fig. 2:**
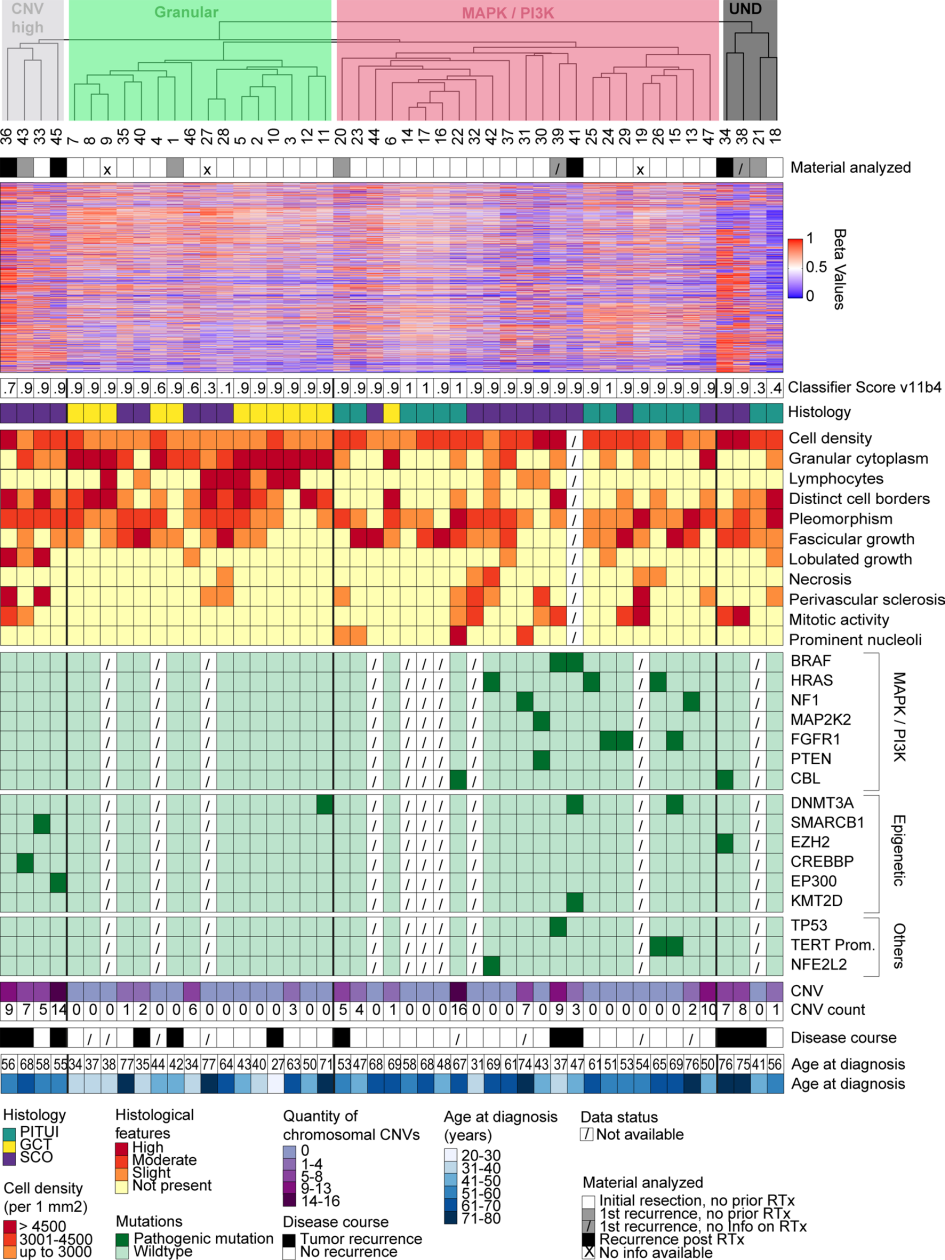
Unsupervised hierarchical clustering of DNA methylation data in comparison to clinical and molecular data. The analysis indicates two main methylation groups, one small group, and four undefined cases. The largest group is enriched for spindle cell oncocytomas and pituicytomas and strongly enriched for mutations of the MAPK/PI3K pathway (MAPK/PI3K group, red). The 2nd largest group is enriched for granular cell tumors (Granular group, green). This group shows a paucity of copy number changes and rare oncogenic mutations. A small group consists of four cases with high numbers of copy number changes and frequent epigenetic regulator mutations (CNV high group, grey). *RTx* radiotherapy, *UND* undefined, *CNV* copy number variation, *PITUI* pituicytoma, *GCT* granular cell tumor, *SCO* spindle cell oncocytoma

Evaluation of histological features was performed on H&E stained slides which were available for 46 of 47 cases (original institutional diagnosis *n* = 12 granular cell tumor (Fig. 1a); *n* = 14 pituicytoma (Fig. 1b); *n* = 21 spindle cell oncocytoma (Fig. 1c and d)). Several cases exhibited increased cell density (Fig. 1e left), lobulated growth (Fig. 1e right), increased nuclear pleomorphism (Fig. 1f left) or extensive epithelioid cytology (Fig. 1f right). Cytoplasmic granularity was seen in varying degrees in tumors diagnosed as spindle cell oncocytoma (Fig. 1c and d). The graded histological features are listed with clinical and molecular data for individual cases in Fig. 2. As expected from the literature, spindle cell oncocytomas showed a particularly high variability of morphological features. Pituicytomas and spindle cell oncocytomas showed overlapping features in many tumors based on H&E evaluation.

### Molecular evaluation

For the molecular characterization of posterior pituitary tumors, we performed targeted next-generation DNA sequencing (*n* = 37), genome-wide DNA methylation profiling (*n* = 47), and chromosomal copy number analysis from DNA methylation data (*n* = 47).

#### Spindle cell oncocytoma and pituicytoma show recurrent MAPK/PI3K pathway and epigenetic regulator mutations

We performed next-generation sequencing using the UCSF500 Cancer Gene Panel. Sufficient material was available for 37 cases (10 granular cell tumors, 18 spindle cell oncocytomas, 9 pituicytomas). We detected 27 likely pathogenic variants in 17 of the 37 cases (Table 1 and Fig. 2). Fourteen of the variants were in genes within the MAPK and PI3K signaling pathways, and many of these are known cancer driving mutations. Two tumors harbored the activating p.V600E hotspot mutation in *BRAF*, three tumors harbored the activating p.N546K or p.K656E hotspot mutations in *FGFR1*, two tumors harbored activating hotspot mutations at codon p.Q61 in *HRAS*, and another tumor harbored a potentially pathogenic mutation in *HRAS* at p.P169. Two tumors harbored recurrent hotspot missense mutations in the *CBL* oncogene (one of which is a major hotspot in dozens of myeloid neoplasms and other tumor types [p.Y371C], and the other of which is a minor hotspot in a few myeloid neoplasms and other tumors [p.P495Q]), two tumors harbored mutations in the *NF1* tumor suppressor gene (one with two different truncating mutations and the other with a potentially pathogenic missense mutation), and one harbored both a start-loss mutation in the *PTEN* tumor suppressor gene and a missense mutation in the *MAP2K2* oncogene (which encodes MEK2) at a codon recurrently targeted by somatic mutation in melanomas and other tumor types [45]. Seven tumors harbored truncating frameshift, nonsense, or splice site variants or known pathogenic missense mutations in genes involved in epigenetic regulation including histone tail modification (*EP300*, *CREBBP*, *EZH2*, *KMT2D*), chromatin remodeling (*SMARCB1*), or DNA methylation (*DNMT3A*). Two tumors harbored the c.-124C > T hotspot mutation in the promoter region of the *TERT* gene known to activate telomerase gene expression. One tumor harbored a known deleterious missense mutation in the *TP53* tumor suppressor gene, and another tumor harbored a small in-frame deletion in the *NFE2L2* gene (also known are NRF2), which affected a known mutational hotspot in the gene recurrently targeted by missense variants or similar small in-frame indels in squamous cell carcinomas of the lung, esophagus, and oropharynx. We also searched for in-frame fusions involving the genes on the panel with intronic regions tiled for detection of structural rearrangements (e.g. *BRAF*, *RAF1*, *FGFR1-3*, *NTRK1-3*, *MET*, *ALK*, *ROS1*, *MYB*, *MYBL1*, *RELA*), but did not detect any. No focal amplifications or deep deletions were observed in any of the investigated tumors. In 20 of the tumors, no likely pathogenic variants were identified by the targeted sequencing assay.

**Table 1:** Likely pathogenic single nucleotide variants and indels identified in the 37 posterior pituitary tumors analyzed by targeted next-generation DNA sequencing

Histologic type	Case ID	Detected genomic variant
Granular cell tumor	1	None
	2	None
	3	None
	5	None
	6	None
	7	None^#^
	8	None^#^
	10	None
	11	DNMT3A c.2408 + 2 T > G
	12	None
Pituicytoma	13	NF1 p.S2549*, NF1 p.R997fs
	15	FGFR1 p.N546K, DNMT3A p.P256fs, TERT c.-124C > T
	18	None
	20	None
	22	CBL p.Y371C
	23	None
	24	FGFR1 p.N546K
	25	HRAS p.Q61R
	26	HRAS p.Q61K, TERT c.-124C > T
Spindle cell oncocytoma	29	FGFR1 p.K656E
	30	MAP2K2 p.F57I, PTEN p.0?
	31	NF1 p.H389Y
	33	SMARCB1 p.A163fs
	34	CBL p.P495Q, EZH2 p.Y646N
	36	None
	37	None
	38	None
	39	BRAF p.V600E, TP53 p.V218E
	41	BRAF p.V600E, DNMT3A p.P904L, KMT2D p.V1674fs
	42	*HRAS p.P169L*, NFE2L2 p.78_79del
	43	CREBBP p.S318_V319delins*
	45	EP300 p.M136fs
	47	None
	35^**×**^	None
	40^**×**^	None
	46^**x**^	None
	28^**×**^	None

Potentially pathogenic variants are written in italics

^#^Low sequencing coverage was obtained for these samples that may have impeded the ability to detect pathogenic mutations in genes targeted for sequencing

^**×**^SCOs clustering with granular DNA methylation group

Interestingly, the distribution of identified genetic alterations was clearly not random among the different histologic types of posterior pituitary tumors (Fig. 2). Within the 10 sequenced granular cell tumors, we only detected a single case with a driver variant (in the epigenetic regulator *DNMT3A*). In contrast, driver variants were frequently identified in pituicytomas (10 variants in 9 cases) and spindle cell oncocytomas (14 variants in 18 cases). In the 4 sequenced spindle cell oncocytomas falling in the granular DNA methylation group by clustering, no mutations were observed, comparable to the low frequency of detectable oncogenic alterations in granular cell tumors. Mutations of MAPK/PI3K pathway genes were observed at high rates in both pituicytomas (66%, 6 of 9 cases) and spindle cell oncocytomas (38%, 7 of 18 cases), but were not observed in any of the granular cell tumors. Epigenetic regulator mutations were observed in all histologic types but were enriched in spindle cell oncocytomas (spindle cell oncocytoma: 6 variants in 18 cases; granular cell tumor: 1/10; pituicytoma: 1/9). The two tumors with *TERT* promoter mutations both had accompanying MAPK/PI3K pathway mutations present (one with *HRAS* p.Q61K and one with *FGFR1* p.N546K) (Table 1, Fig. 2). Detailed information on all detected variants is provided in Supplementary Table 3.

In two of the evaluated tumors (case ID 39 and 41), the genetic data indicated the presence of intratumoral heterogeneity. In tumor case 39, *BRAF* p.V600E mutation was present at a clonal allele frequency of 35%, indicating that it was likely to have been acquired as an early event during tumorigenesis and to be present in the majority of tumor cells in the heterozygous state. In contrast, *TP53* p.V218E mutation was found in this tumor at a subclonal allele frequency of 13%, indicating that it was likely to have been acquired later during tumor development and therefore only present in a subset of tumor cells. In tumor case 41, *BRAF* p.V600E and *DNMT3A* p.P904L mutations were both present at clonal allele frequencies of 32%, indicating their acquisition early during tumorigenesis and presence in the majority of tumor cells. In contrast, *KMT2D* p.V1674fs mutation was found in this tumor at a subclonal allele frequency of 5%, indicating its presence in only a subset of tumor cells.

#### DNA methylation profiling suggests two main subgroups of posterior pituitary tumors

We next performed a tSNE analysis of our 47 cases together with the reference cohort of the brain tumor classifier (Supplementary Fig. 1) [7]. This analysis demonstrated that all but two tumors of our cohort formed a well-defined group distinct from the other 81 tumor classes.

Calculation of DNA methylation based classification scores (classifier version v11b4 [7]) indicated that for all tumors, the highest score was of the DNA methylation class pituicytoma/granular cell tumor/spindle cell oncocytoma, but seven did not reach the cutoff of 0.9 for a valid classification (Fig. 2). The classifier score provides information on the degree of similarity or match of a methylation profile to a specific methylation class. It ranges from 0 [no similarity] to 1 [highest similarity] [7]. Nonetheless, the histology of these seven tumors closely aligned with that of the posterior pituitary tumor spectrum, so we kept these cases as part of this series. Two aberrant cases from the above tSNE analysis showed particularly low classification scores (Fig. 2). Both of these cases showed abundant lymphoplasmacytic infiltrates (Supplementary Fig. 2a, b), potentially explaining the lower classification score. For the other 5 cases (case IDs 4, 18, 21, 36, 46), no particular reason for the low classification scores could be identified. Three of these tumors were primary lesions without prior treatment, one tumor was a biopsy of a recurrent tumor without prior treatment, and one tumor was a second recurrence following prior radiotherapy.

We next performed an unsupervised hierarchical clustering of the DNA methylation data of the 47 posterior pituitary tumors (Fig. 2). Histological, sequencing, and copy number data is displayed alongside this unsupervised clustering data. The clustering suggested the separation into two large groups, one possible small group, and four outlier cases (see below). The groups were provisionally named according to their most prominent molecular feature: the largest DNA methylation-clustering group was characterized by a high rate (*n* = 12/17; 71%) of MAPK/PI3K pathway alterations (MAPK/PI3K group) and included cases mainly from the morphological spectrum of pituicytoma and spindle cell oncocytoma. The mean patient age at diagnosis in this group was 58 years (range 31–76 years), with a slight male predominance (female:male ratio 1:1.5). The second largest group was dominated by granular cell tumors (Granular group: 11 granular cell tumors, 5 spindle cell oncocytomas). For 4 of the 5 spindle cell oncocytomas falling into this group (case ID 35, 46, 27, 28), material for additional immunostaining for mitochondrial antigen was available. All cases showed extensive cytoplasmic labeling compatible with the diagnosis of spindle cell oncocytoma (Supplementary Fig. 3). The tumors in the Granular group were mostly devoid of identifiable pathogenic mutations and showed no or very few copy number changes. The mean patient age at diagnosis in this group was 42.5 years (range 27–77 years) and the female:male ratio was 1:0.6. We further observed a small group of four cases with abundant copy number alterations (CNV high group: 4 spindle cell oncocytomas). This small group was enriched for epigenetic regulator mutations (*n* = 3/4; 75%) and did not show MAPK/PI3K pathway alterations. The mean patient age was 57 years (range 55–68 years) and female:male ratio was 4:0.

Four cases (ID 18, 21, 34, 38) did not fall into the above groups but rather represented outliers that we did not consider to form a separate group. They are designated as “undefined” (UND) in Fig. 2 and may represent a collection of rare additional tumor types related to the posterior pituitary. Cases 34 and 38 were diagnosed as spindle cell oncocytomas but had unusual hypercellularity (Supplementary Fig. 2c, d). Case 38 further showed an increased mitotic count of up to 3 mitotic figures per 10 HPF. Cases 18 and 21 were diagnosed as pituicytomas and we did not identify specific unifying histomorphological features.

**Fig. 3:**
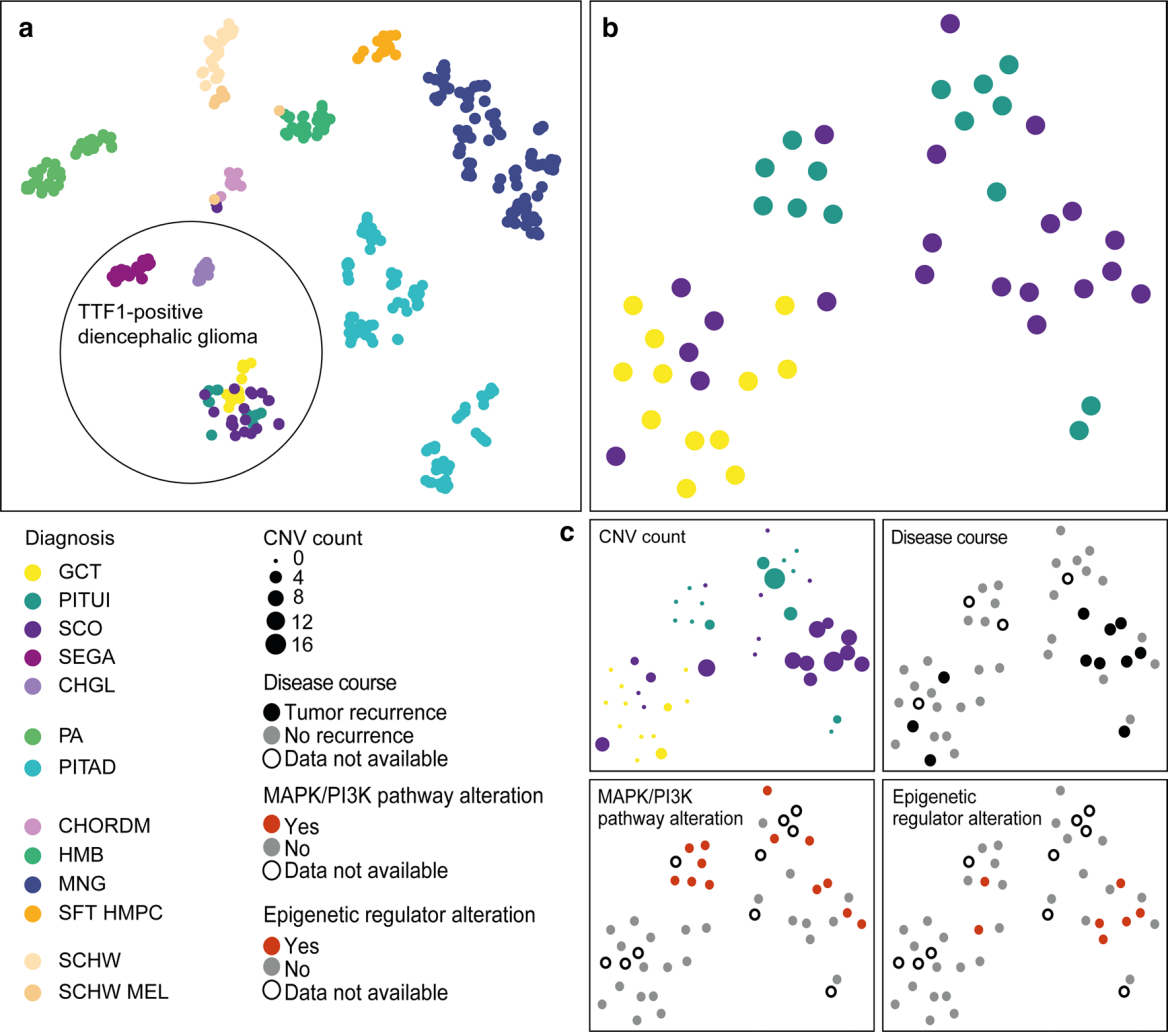
Depiction of *t* distributed stochastic neighbor embedding (tSNE) of selected groups of the brain tumor classifier cohort [7] together with the cases of this series. The tumors of this study all group together closely and separate well from the other included groups, in particular from other TTF1-positive glioma types. Two cases (case 27 and 28) fall slightly to the side together with two cases of other groups. (see Supplementary Fig. 1). *PITUI* pituicytoma, *GCT* granular cell tumor, *SCO* spindle cell oncocytoma, *SEGA* subependymal giant cell astrocytoma, *CHGL* chordoid glioma of the third ventricle, *PA* pilocytic astrocytoma, *PITAD* pituitary adenoma, *CHORDM* chordoma, *HMB* hemangioblastoma, *MNG* meningioma, *SFT HMPC* solitary fibrous tumor/hemangiopericytoma, *SCHW* schwannoma, *SCHW MEL* melanotic schwannoma (**a**). tSNE plots based on Pearson correlation distance matrix of posterior pituitary tumors illustrates a slight separation of granular tumors in the lower left from pituicytomas and spindle cell oncocytomas on the right (**b**). When the NGS data is overlaid, the MAPK/PI3K pathway and epigenetic regulator mutations predominantly occurred in the large group dominated by spindle cell oncocytomas and pituicytomas (**c**). When overlaid with the copy number information (**c**), it was evident that most cases with high rates of copy number changes grouped together and that the cases with high copy number load were partially the same cases later showing recurrence (**c**) Missing data is indicated by open circles

tSNE dimensionality reduction of the 47 posterior pituitary tumors together with selected tumor classes from the brain tumor classifier cohort [7] demonstrated a grouping of posterior pituitary tumors compared to the other classes (Fig. 3a). Interestingly, the closest neighbors of posterior pituitary tumors were the other two TTF1 expressing tumor groups SEGA and chordoid glioma. In a tSNE analysis of only posterior pituitary tumors, a slight separation of granular cell tumors was observed whereas most of the spindle cell oncocytomas and pituicytomas grouped together in one large group (Fig. 3b). When the NGS data are overlaid, the MAPK/PI3K pathway and epigenetic regulator mutations all occurred in the large group dominated by spindle cell oncocytomas and pituicytomas (Fig. 3c). When overlaid with the copy number information (Fig. 3c), it was evident that most cases with high rates of copy number changes grouped together and that the cases with high copy number load were partially the same cases later showing recurrence (Fig. 3c).

#### Analysis of differentially methylated regions

In contrast to most other brain tumors, the tumors of this study only occur in a single location, the posterior pituitary lobe or the connection to the hypothalamic region termed the “infundibulum”. This allows a direct comparison of tumor DNA methylation profiles to the potential tissue of origin. In both tSNE analysis and hierarchical clustering of DNA methylation data, we observed a close clustering of posterior pituitary tumors and normal posterior lobe/infundibulum (Supplementary Fig. 4). To have a more exact measure of how different the two main methylation groups are from the normal tissue, we calculated the number of differentially methylated regions (DMR) with a mean beta value difference of at least 0.2. The potential third group (CNV high) was too small to be included in this analysis. This resulted in the finding that the MAPK/PI3K group shows remarkably little difference to normal posterior pituitary tissue (only 16 DMR). In contrast, the Granular group harbored 69 DMRs compared to normal posterior pituitary. For cases with additional information available, annotation of the DMRs was done using the public databases Ensembl, Reactome, and Gene Ontology (Supplementary Tables 4 and 5). While it is not possible to directly infer gene expression changes from methylation data, we categorized the DMR as likely inhibitory, possible inhibitory, or unknown concerning expected effect on gene expression. It was of interest that among the 69 DMR of the Granular group, 6 of the affected genes were associated with G protein-coupled receptor signaling or signaling by receptor tyrosine kinases (Supplementary Table 4). This was not observed among the 16 DMR of the MAPK/PI3K group (Supplementary Table 5).

We further calculated the number of DMR between the MAPK/PI3K group and Granular group with the same cutoff as above. This indicated 50 DMRs between the two main methylation groups (data not shown). To compare these groups, we performed the same DMR calculations for the brain tumor reference classes midline pilocytic astrocytoma vs posterior fossa pilocytic astrocytoma and identified 133 DMR (data not shown) and for midline pilocytic astrocytoma vs. pleomorphic xanthoastrocytoma we identified 1304 DMR (data not shown).

#### Copy number analysis

Copy number profiles were determined from both NGS and DNA methylation array data, which showed highly concordant results (data not shown). For copy number changes inferred from NGS data see Supplementary Table 6.

**Fig. 4:**
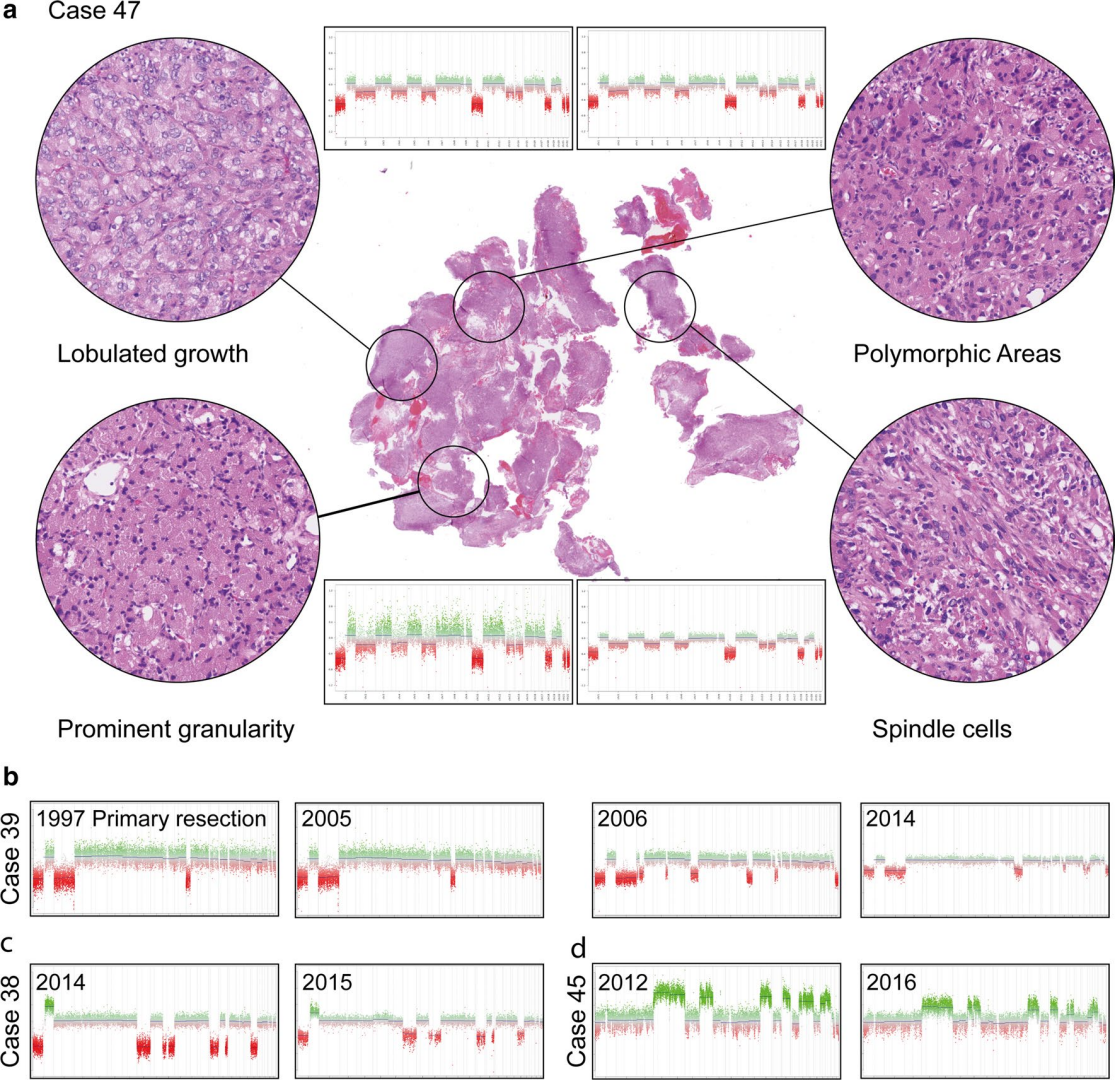
Illustration of the stability of copy number changes within a selected sample (case 47, 10 CNVs) with four distinct morphological areas. Copy number changes are identical in all four areas (**a**). Illustration of copy number plots of three cases with tissue from two or more operation time points (**b**–**d**). In two of the cases single chromosomal changes were gained or lost over time, whereas most aberrations remained stable

In 27/47 cases, we detected no chromosomal changes. We observed one or more chromosomal gains or losses in 61% of spindle cell oncocytoma (13/21), 35% of pituicytoma (5/14), and 17% of granular cell tumors (2/12). The total number of copy number changes per case was clearly higher in spindle cell oncocytoma with 10/21 cases (47%) showing 5 or more chromosomal changes. This occurred in only 2/14 (14%) pituicytomas, and none of the granular cell tumors. Further, the type of chromosomal aberrations differed with the gains and losses always affecting whole chromosomes or whole chromosome arms (breakpoint at/near the centromere) in granular cell tumors and pituicytomas but also occurring within a chromosome arm in spindle cell oncocytoma. Focal amplifications or focal deep/homozygous deletions were not observed in any of the 47 tumors. To investigate the stability of copy number changes within different regions of a sample we selected a case (ID 47, 10 CNVs) with four distinct morphological areas and separately performed DNA methylation and copy number analyses. In all four areas, the copy number changes were identical (Fig. 4a). We further investigated three cases with tissue from two or more time points (primary and recurrence). All cases harbored chromosomal copy number aberrations at initial resection (Case 39, Fig. 4b; Case 38, Fig. 4c, Case 45, Fig. 4d). In two of the cases single chromosomal changes were gained or lost over time, whereas most aberrations remained stable.

Supplementary Fig. 5 shows summary copy number plots for the three methylation clustering groups illustrating the fraction of tumors belonging to the group harboring chromosomal gains/losses. Both the Granular and the MAPK/PI3K group did not demonstrate any highly recurrent copy number alterations. The small epigenetic group of CNV high tumors shows numerous chromosomal alterations, with all four cases showing a loss of chromosome 1p and two cases showing additional gains of 1q, as well as other recurrent chromosomal alterations.

### Patient outcome analysis

#### Tumors with spindle cell oncocytoma morphology and chromosomal copy number imbalances showed the highest rate of recurrence

Outcome data was available for 37 of the 47 patients of the series. Seven patients died during follow up: four patients died of unknown reason, one patient died after cerebral bleeding of unknown reason (no prior radiotherapy), one patient died from an occlusion of the internal carotid artery and subarachnoid hemorrhage one month after repeated tumor surgery (prior radiotherapy), and another patient died 5 days after tumor associated bleeding and occlusion of the internal carotid artery (no prior radiotherapy). As none of the patient deaths was clearly attributable to tumor biology, we only performed outcome analysis for progression free survival.

**Fig. 5:**
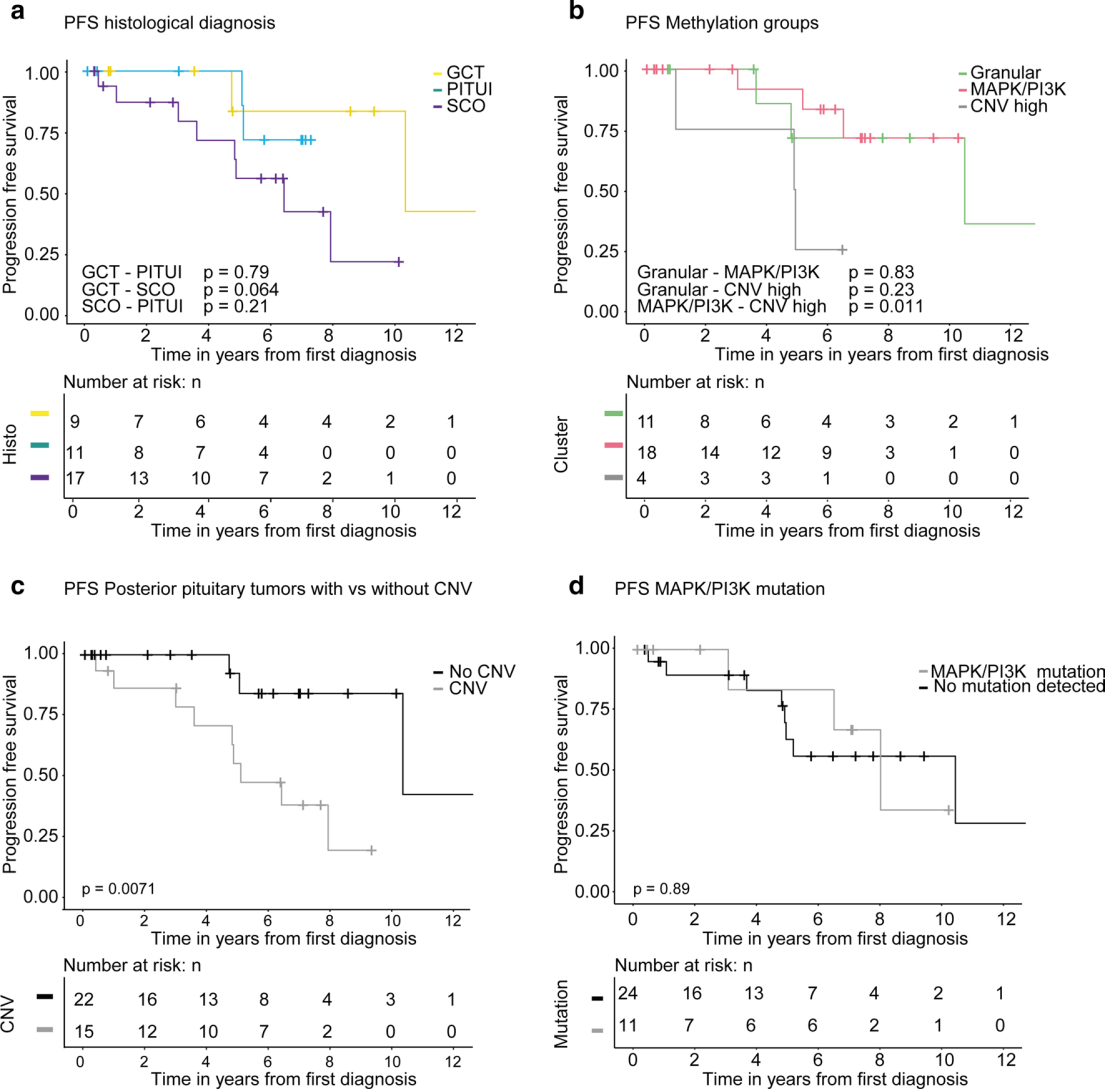
Kaplan–Meier analysis of progression free survival. Analysis stratified by central histological diagnosis. Spindle cell oncocytomas relapsed more frequently and earlier compared to the two other groups. Statistical significance was not reached for spindle cell oncocytoma vs pituicytoma (*p* = 0.21) or for the other groups (**a**). Analysis according to DNA methylation subgroups shows shorter progression free survival for CNV high group vs. the MAPK/PI3K group (*p* = 0.011) with the granular group being in-between these two (not significant) (**b**). Grouping of all posterior pituitary tumors according to presence of copy number alterations (i.e. any chromosomal gains or losses) showed that tumors with any copy number variation had shorter progression free survival (*p* = 0.0071) (**c**). Analysis according to presence of any mutation of MAPK/PI3K pathway vs no detected mutation did not reach statistical significance (*p* = 0.89) (**d**)

We performed univariate progression free survival analysis for central histological diagnosis, DNA methylation clusters, presence of no vs any copy number alteration, and presence of MAPK/PI3K mutation vs no detected mutation in the respective pathway (Fig. 5a–d).

Recurrence rates differed between the three main histological diagnoses. Spindle cell oncocytomas showed the highest rates of recurrence with 8/21 (38%) cases requiring a second surgery with a median time to recurrence of 4.8 years (range 5 months to 9.4 years). Only two granular cell tumors relapsed (time to recurrence 4.7 years and 10.3 years) and two of the pituicytomas (time to recurrence 5.1 years and 5 years). In Kaplan–Meier analysis there was a trend for worse outcome of spindle cell oncocytoma compared to pituicytoma, but this did not reach statistical significance (*p* = 0.21; Fig. 5a).

When analyzed for the three DNA methylation groups, the “CNV high” group had shortest progression free survival with a statistically significant difference to the MAPK/PI3K group (*p* = 0.011, Fig. 5b).

We further analyzed if copy number alterations were associated with progression free survival. Among the whole cohort, patients with tumors showing chromosomal imbalances had a worse progression free survival (*p* = 0.0071; Fig. 5c). This association with progression free survival was still observed when excluding all cases where the initial tumor was not available for analysis (*p* = 0.05 [all recurrences and unclear cases excluded], Supplementary Fig. 6a, *p* = 0.02 [only recurrences with prior radiation therapy excluded], Supplementary Fig. 6b).

No association of the mutation status and disease progression was observed when comparing mutation of the different pathways vs wildtype (data shown for MAPK/PI3K mutation, *p* = 0.89, Fig. 5d).

#### Integrated histomolecular classification improves separation of posterior pituitary tumors into prognostic groups

The presence of any versus no copy number alteration seemed to be the most relevant factor for separating posterior pituitary tumors into prognostic groups based on our tumor cohort (Fig. 5c). Further, pituicytoma and spindle cell oncocytoma clearly form a histological continuum and our molecular data indicates that both tumors share a common methylation profile and have a common spectrum of mutations. Granular cell tumors in contrast are both distinct in histology and molecular pathogenesis.

**Fig. 6:**
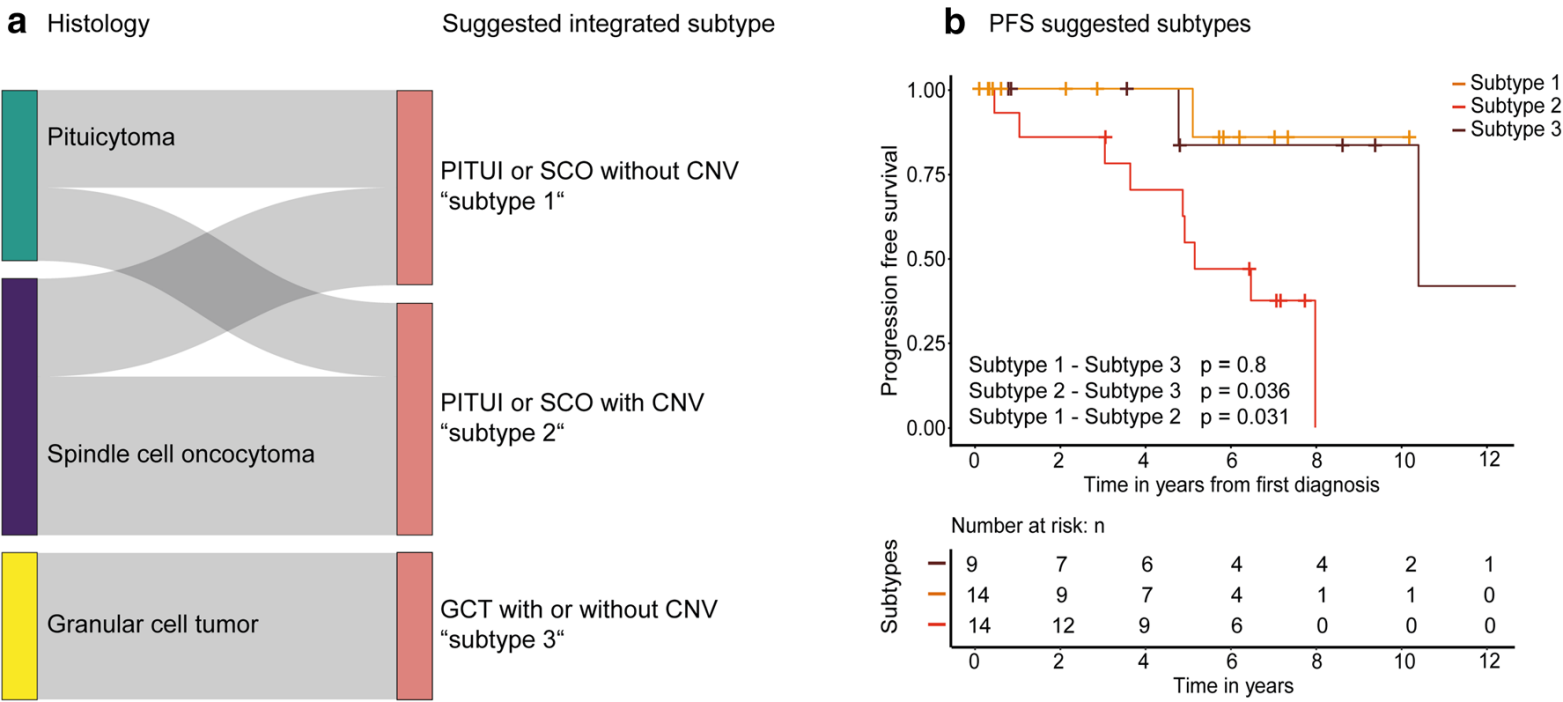
A sankey plot for re-grouping of the posterior pituitary tumors of this series in the 3 suggested subtypes shows mainly shifting of 8 spindle cell oncocytomas without CNV to subtype 1 and shifting of 6 pituicytomas with CNV to subtype 2 (**a**). Kaplan–Meier analysis of progression free survival stratified by the three proposed subtypes shows improved separation compared to stratification based on histologic diagnosis (**b**)

Based on these findings we propose an integrated histomolecular classification of posterior pituitary tumors into 3 subtypes (Fig. 6):

Subtype 1 includes tumors of pituicytoma or spindle cell oncocytoma morphology without any copy number alteration (*n* = 16). These tumors are most likely to harbor mutations of MAPK/PI3K pathway, epigenetic regulators, or other pathways. They usually follow a benign clinical course. Only 1 tumor belonging to this group recurred during follow up, which was a pituicytoma without copy number alterations and without material available for DNA sequencing analysis. It is of interest that this group included 9 spindle cell oncocytomas that did not recur during median follow up period of 2.8 years. Thus, this group likely indicates spindle cell oncocytomas with low risk of recurrence (Fig. 6a). Subtype 2 (*n* = 19) includes tumors of pituicytoma or spindle cell oncocytoma morphology with presence of chromosomal copy number alterations. 47% (8/17 cases with follow up data) of the tumors in this group recurred during a median follow up period of 7 years. Among those tumors was the only other pituicytoma in this study that recurred during follow up. It harbored 5 CNV and no detectable driver mutation. Subtype 3 is composed of granular cell tumors with or without copy number alterations (*n* = 12). Only 2 tumors of this group recurred during median follow up of 4.7 years and those did not harbor any copy number alterations. Only a single tumor in this group contained an identifiable pathogenic variant (in an epigenetic regulatory gene).

Kaplan–Meier analysis of tumors stratified into these 3 integrated subtypes shows increased separation of the tumors into prognostic groups compared to histological subtypes alone, mutation status alone, or DNA methylation groups alone (*p* = 0.031; Fig. 6b).

## Discussion

**Fig.7:**
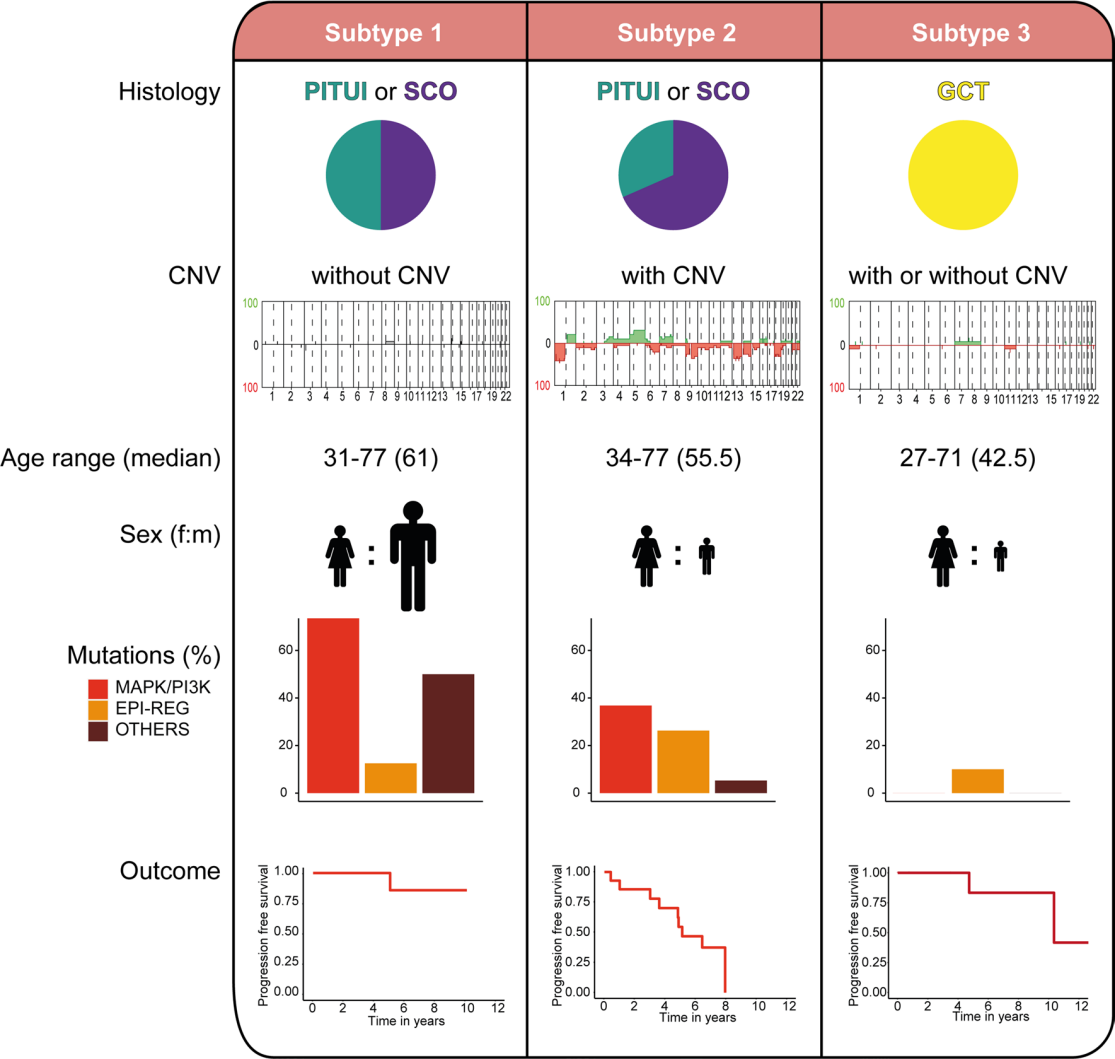
Pictogram summarizing the relation of histological groups, mutation frequencies, chromosomal imbalances, and outcome of the three suggested histomolecular subtypes

We here describe the presence of two main molecular groups among posterior pituitary tumors that partially overlap with the established histological classes. While these new groups show only subtle differences of genome-wide DNA methylation, they vary considerably concerning mutation spectrum and rate of chromosomal imbalances. One of these groups can be further separated by the presence of copy number alterations into tumors with low versus high recurrence rates. Hence, we propose the integrated histo-molecular classification of posterior pituitary tumors into 3 clinically meaningful subtypes (Fig. 7).

DNA methylation differences among tumors of the posterior pituitary are only subtle. Between the two main molecular groups, only 50 DMR were detected in our analyses. When compared to other tumor classes, this is for example lower than the differences observed between midline and posterior fossa pilocytic astrocytomas (133 DMR). This strongly suggests that posterior pituitary tumors have a common cell of origin. Accordingly, the current version of the brain tumor classifier does not separate posterior pituitary tumor subtypes and the subtle differences we found make it likely that a diagnostic DNA methylation based separation will not be possible in such a broad classification tool. Interestingly, in our comparison of DNA methylation data of posterior pituitary tumors with normal posterior pituitary tissue, posterior pituitary tumors enriched for MAPK/PI3K alterations (MAPK/PI3K group) harbored as little as 16 DMR and are thus almost indistinguishable from normal tissue. Tumors from the Granular group harbored slightly more (69 DMR) but were still surprisingly similar to normal tissue. This indicates that the methylation profile of tumors of the main groups of posterior pituitary tumors is almost identical to adult posterior pituitary tissue. This intriguing observation may be the first indication that tumors of the posterior pituitary develop from fully differentiated adult tissue and not necessarily from less differentiated cells (e.g., precursor cells) as expected for most other types of brain tumors.

Yet, a clearly non-random distribution of the histological subtypes and mutations among the DNA methylation groups was evident.

The largest DNA methylation group consists of pituicytomas and a large proportion of spindle cell oncocytomas (MAPK/PI3K group, Fig. 2). These tumors have a high rate of MAPK/PI3K pathway alterations in combination with alterations of epigenetic regulators. Single cases or small series of MAPK mutant pituicytoma or spindle cell oncocytoma have recently also been identified by others [13, 43, 55, 60]. These data strongly indicate that activation of the MAPK/PI3K pathway is a common molecular feature in tumors of this group. Our combined histo-molecular data indicate that tumors of the morphological spectrum of pituicytoma and spindle cell oncocytoma may be best considered as a single group.

Despite our extensive sequencing attempts using a large 500 gene panel, we were not able to identify any significant driver genetic alterations for tumors of the second largest DNA methylation group. This group consisted mostly of granular cell tumors and spindle cell oncocytomas (Fig. 2). Because of the homogenous histological appearance of most cases, a common genetic driver seems likely and these cases may be good targets for more extensive genetic sequencing studies. Five of the 16 cases of this DNA methylation group had not been histologically classified as granular cell tumors (institutional pathologic diagnosis for all five tumors was spindle cell oncocytoma). In line with the other cases of the granular group, none of these cases had any genetic alterations detected by our sequencing approach. This finding suggests that these five cases differ molecularly from other spindle cell oncocytomas. For four of these cases, we performed immunohistochemistry for anti-mitochondrial antigen and all cases showed clear cytoplasmic reactivity as would primarily be expected for spindle cell oncocytomas. Thus these tumors are molecularly closer to granular cell tumors but histologically closer to spindle cell oncocytomas and we currently cannot fully resolve the assignment for these cases. Of the 16 tumors in the Granular group, only four harbored copy number alterations. Interestingly, three of these four tumors were histologically classified as spindle cell oncocytomas. As discussed above, the comparison of DNA methylation data of the Granular group with normal posterior pituitary indicated 69 DMR. While this seems like very few DMR overall, it can be noted that these genetically mostly silent tumors (very few mutations, very few copy number changes), may harbour somewhat more DMR than tumors of the MAPK/PI3K group (16 DMR). When we inferred the DMR of the Granular group individually, it was apparent that six of the genes associated with the DMR were involved in G protein-coupled receptor signaling or signaling by receptor tyrosine kinases (Supplementary Table 4). Further study is required to investigate if these methylation changes also affect gene expression. Still, it is interesting to speculate that possibly the Granular group may be driven by epigenetic alterations of MAPK signaling rather than by genetic mutations of this pathway.

We further identified a small fraction of four spindle cell oncocytomas that formed a putative additional molecular group harboring high numbers of copy number alterations and epigenetic regulator mutations without MAPK alteration (labeled CNV high group, Fig. 2). Two of the cases of this group further showed an unusual histological growth pattern with prominent lobulation and prominent intraparenchymal sclerosis. More cases are required to further define if these cases indeed represent an additional rare tumor subgroup and if these cases have a recognizable histological appearance.

One of the most important findings of this study is the observation of considerable rates of MAPK/PI3K pathway alterations in tumors of either pituicytoma or spindle cell oncocytoma morphology. Several of the here identified MAPK pathway alterations have shown to be targetable by specific therapeutic agents in other entities and diagnostic testing for these alterations seems advisable when additional treatment options are being considered. First cases of treatment success of posterior pituitary tumors by targeted agents have even been reported [13, 55].

Interestingly, we were not able to identify a more uniform genetic driver (or even a more uniform signaling pathway) that was recurrently altered across all tumors. Unlike chordoid gliomas that all have *PRKCA* mutation [17], papillary glioneuronal tumors that all have *PRKCA* fusion [6, 24], SEGA's that all have *TSC1*/2 inactivating mutations [5, 10], gangliogliomas and pilocytic astrocytomas that almost uniformly have *BRAF* or other MAPK alteration [29, 48, 53], posterior pituitary tumors display a truly remarkable diversity of genetic alterations, including a wide spectrum of different MAPK drivers (e.g. *BRAF*, *HRAS*, *MAP2K2*, *CBL*, *FGFR1*), PI3-kinase drivers (e.g. *PTEN*, *CBL*, *FGFR1*), epigenetic regulators (e.g. *DNMT3A*, *EZH2*, *SMARCB1*, *EP300*, *CREBBP*, *KMT2D*), and others (*TP53*, *TERT* promoter, *NFE2L2*).

Several practical consequences can be derived from our data: first, DNA methylation differences between the posterior pituitary tumors are very subtle, indicating that these tumors may be best classified as subtypes/variants of a single type/entity. DNA methylation profiling may not be suitable for routine identification of subtypes/variants. Second, copy number alterations may be of value for identifying tumors with shorter time to recurrence. This may be of value at first diagnosis to identify cases with need for closer follow up. Future studies may test if these patients may also benefit from adjuvant therapy (e.g. irradiation). Third, a substantial fraction of posterior pituitary tumors harbors potentially targetable MAPK/PI3K alterations. A range of genes within the MAPK/PI3K pathways may be affected and diagnostic testing should account for this. The spindle cell oncocytoma and pituicytoma subtypes are particularly prone to these alterations and should be interrogated at least at recurrence or when additional therapeutic options are required (e.g. residual non-resectable tumor). Fourth, we propose a reclassification of pituicyte-derived tumors into three clinically relevant new subtypes. One group represents granular cell tumors as previously established. The other two groups both represent a mix of spindle cell oncocytoma and pituicytoma histology but are separated by the presence or absence of chromosomal copy number alterations into prognostic groups. We believe this will result in a more reproducible and clinically practical subgrouping of posterior pituitary tumors.

## Supplementary Information

Below is the link to the electronic supplementary material.

Depiction of a t-distributed stochastic neighbor embedding (tSNE) of the brain tumor classifier cohort [7] together with the cases of this series. The colors represent the three histological groups of posterior pituitary tumors and the main tumor classes of the brain tumor classifier. The tumors of this study all group together closely. The inset shows a higher zoom in the posterior pituitary tumors and indicates that all three main histological classes form a single group with no clear separation at the scale of this analysis. Two cases (Case 27 and 28) fall slightly to the side together with two cases of other groups. Both cases had prominent lymphoplasmacytic infiltration, likely contributing to this unusual clustering behavior (see Figure 2 and Supplementary Figure 2) (TIF 22238 KB)

Depiction of Hematoxylin and Eosin histology (a, b, c, d, magnification 200× (a,b) and 400x (c,d)) for cases with unusual grouping in the large tSNE (case 27 and 28; Supplementary Figure 1) or in the clustering analysis (case 38 and 34, Figure 2). In the histological review, cases 27 and 28 showed prominent lymphoplasmacytic infiltrates whereas cases 38 and 34 were remarkable for high cell density (TIF 33541 KB)

Anti-mitochondrial-antigen staining and H&E stain of 4 spindle cell oncocytomas that clustered with the granular group (case 35, a+b; case 28, c+d; case 27, e+f; case 46, g+h; original magnification 400×) (TIF 79994 KB)

tSNE representation with all posterior pituitary tumors of this series and additional normal control tissue, including samples from posterior pituitary lobe and infundibulum. Tumor and control samples group exceptionally close together (TIF 12436 KB)

Summary copy number plots for the three posterior pituitary tumor DNA methylation groups. Granular group tumors and MAPK/PI3K group tumors show no highly recurrent cytogenetic alterations. Eleven of sixteen (69%) granular group tumors and 14 of 23 (60%) of MAPK/PI3K group tumors harbored no detectable chromosomal alterations (flat genome) with the remaining cases showing infrequent chromosomal gains or losses. Amplifications or focal deletions were not observed (a, b). In contrast, the small methylation group “CNV high” was characterized by numerous chromosomal gains and losses including loss of chromosome 1p in all four cases(c) (TIF 35106 KB)

Kaplan-Meier analysis for progression free survival for cases of this series (a) excluding all cases where the initial tumor was not available for analysis or (b) excluding all cases that had a documented radiation therapy prior to sampling. In both subsets the presence of any copy number alterations was still significantly associated with worse outcome (TIF 18465 KB)

Supplementary file7 (XLSX 12 KB)

Supplementary file8 (XLSX 15 KB)

Supplementary file9 (XLSX 15 KB)

Supplementary file10 (XLSX 20 KB)

Supplementary file11 (XLSX 12 KB)

Supplementary file12 (XLSX 11 KB)

## Data Availability

DNA methylation array data are available from the Gene Expression Omnibus (GEO) repository under accession number GSE185041 (https://www.ncbi.nlm.nih.gov/geo/). DNA sequencing results are available in the online supplementary material. Raw sequencing data files are available from the authors upon request.
