# Supplementary figures and images for: Genetic and epigenetic characterization of posterior pituitary tumors
Source: Acta Neuropathol. 2021 Oct 18;142(6):1025–43. doi: 10.1007/s00401-021-02377-1 (PMC8568760; doi:10.1007/s00401-021-02377-1)

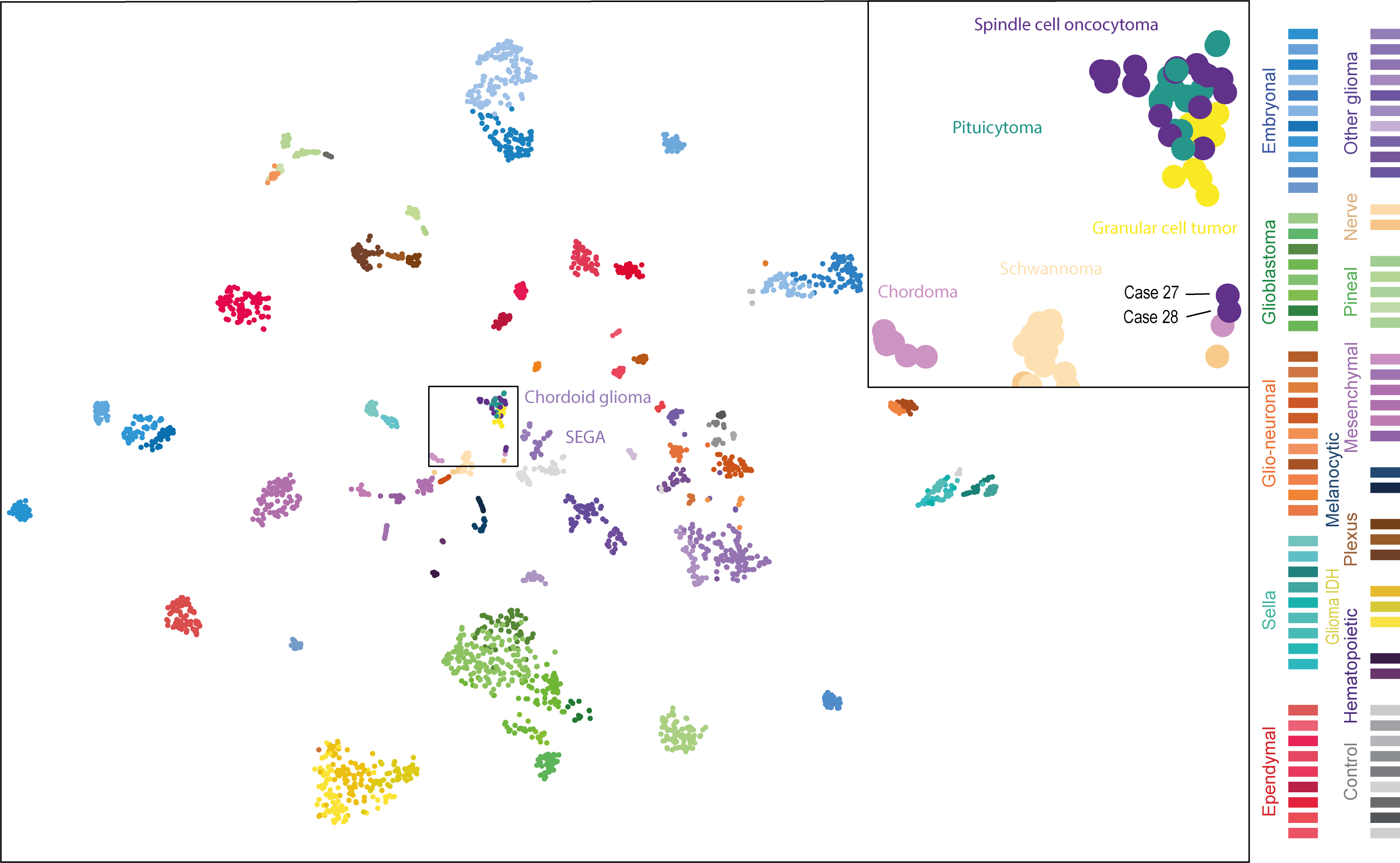

Supplement: Supplementary file 1 — Depiction of a t-distributed stochastic neighbor embedding (tSNE) of the brain tumor classifier cohort [7] together with the cases of this series. The colors represent the three histological groups of posterior pituitary tumors and the main tumor classes of the brain tumor classifier. The tumors of this study all group together closely. The inset shows a higher zoom in the posterior pituitary tumors and indicates that all three main histological classes form a single group with no clear separation at the scale of this analysis. Two cases (Case 27 and 28) fall slightly to the side together with two cases of other groups. Both cases had prominent lymphoplasmacytic infiltration, likely contributing to this unusual clustering behavior (see Figure 2 and Supplementary Figure 2) (TIF 22238 KB) [file 401_2021_2377_MOESM1_ESM.tif]

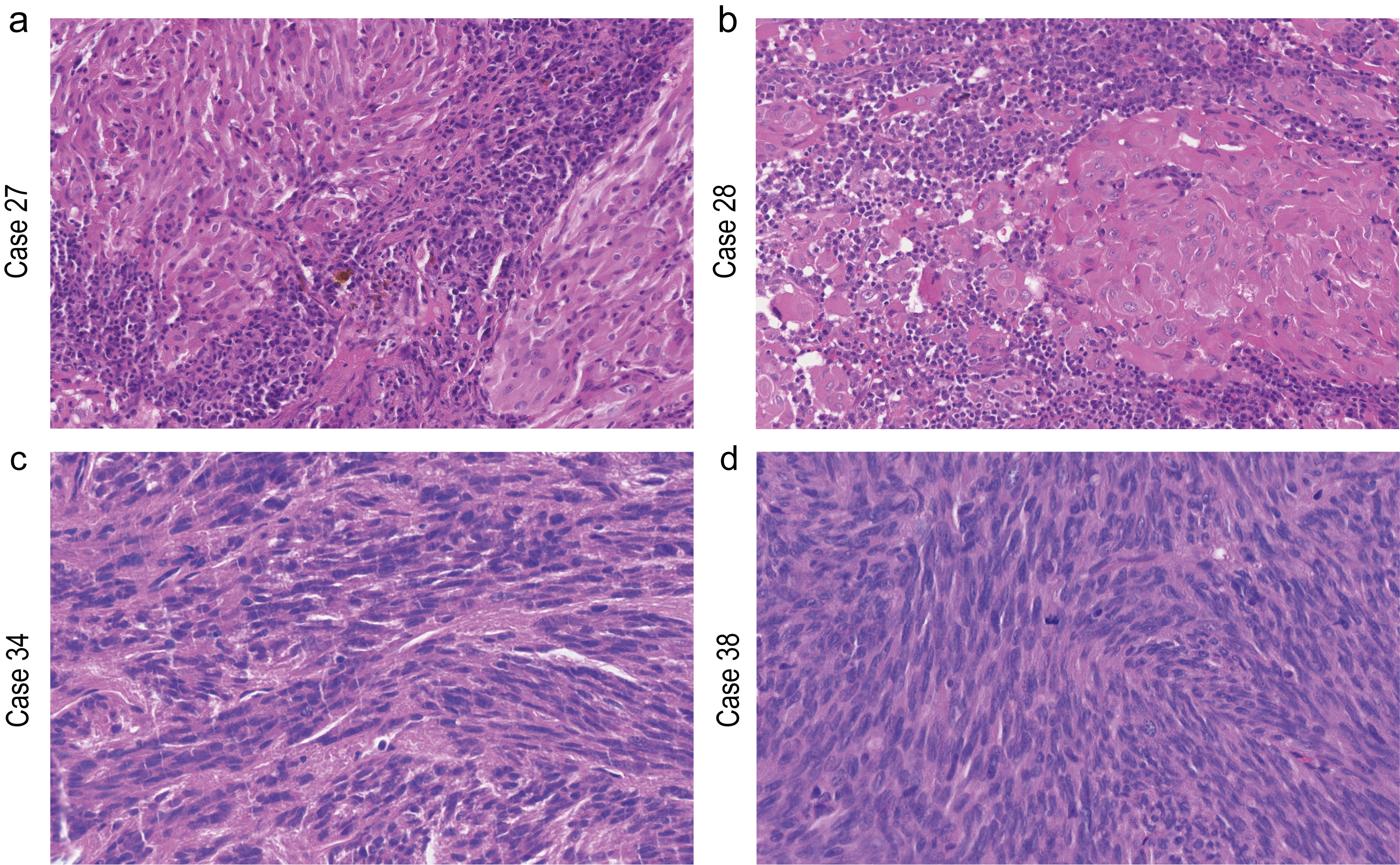

Supplement: Supplementary file 2 — Depiction of Hematoxylin and Eosin histology (a, b, c, d, magnification 200× (a,b) and 400x (c,d)) for cases with unusual grouping in the large tSNE (case 27 and 28; Supplementary Figure 1) or in the clustering analysis (case 38 and 34, Figure 2). In the histological review, cases 27 and 28 showed prominent lymphoplasmacytic infiltrates whereas cases 38 and 34 were remarkable for high cell density (TIF 33541 KB) [file 401_2021_2377_MOESM2_ESM.tif]

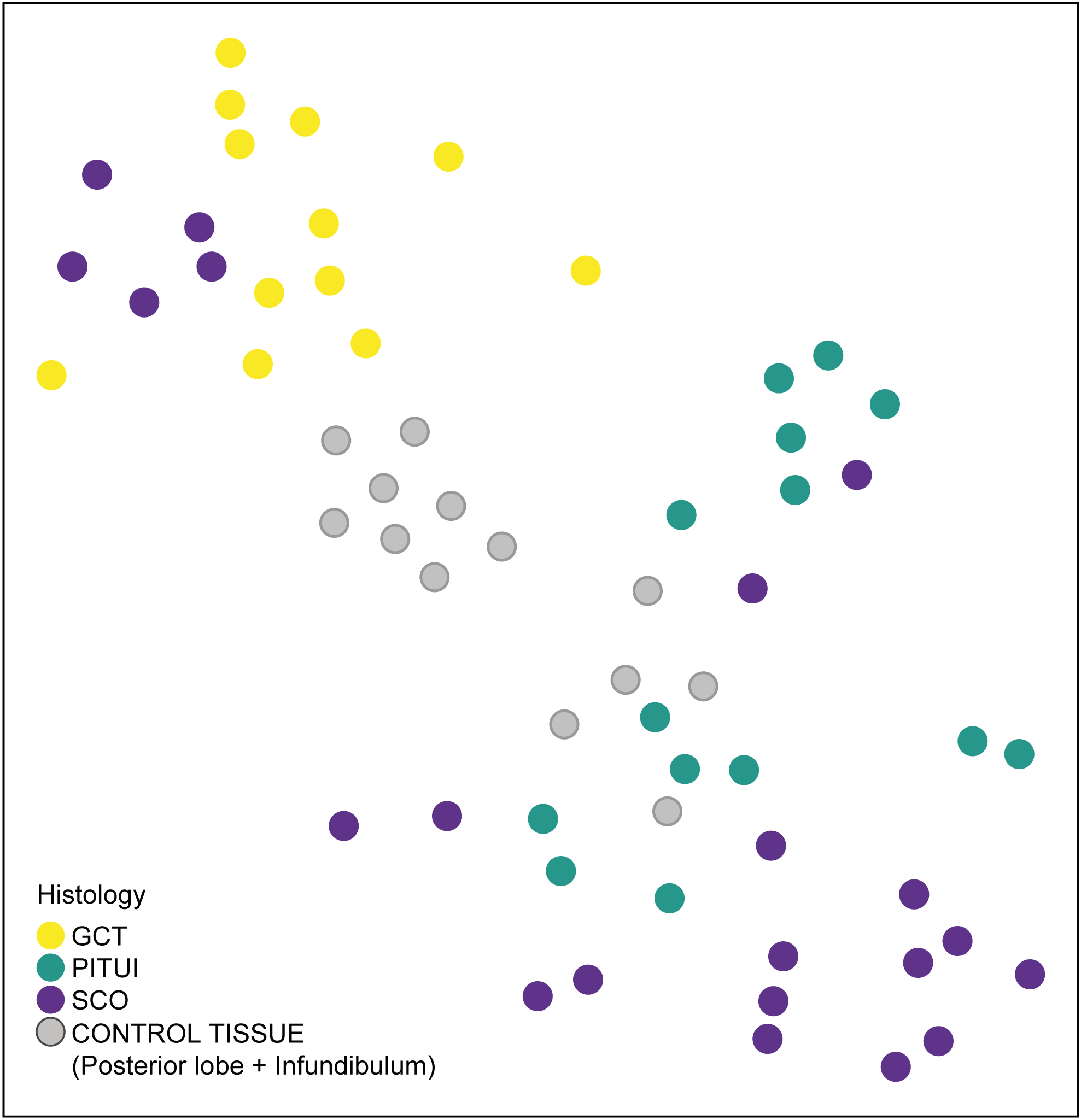

Supplement: Supplementary file 4 — tSNE representation with all posterior pituitary tumors of this series and additional normal control tissue, including samples from posterior pituitary lobe and infundibulum. Tumor and control samples group exceptionally close together (TIF 12436 KB) [file 401_2021_2377_MOESM4_ESM.tif]

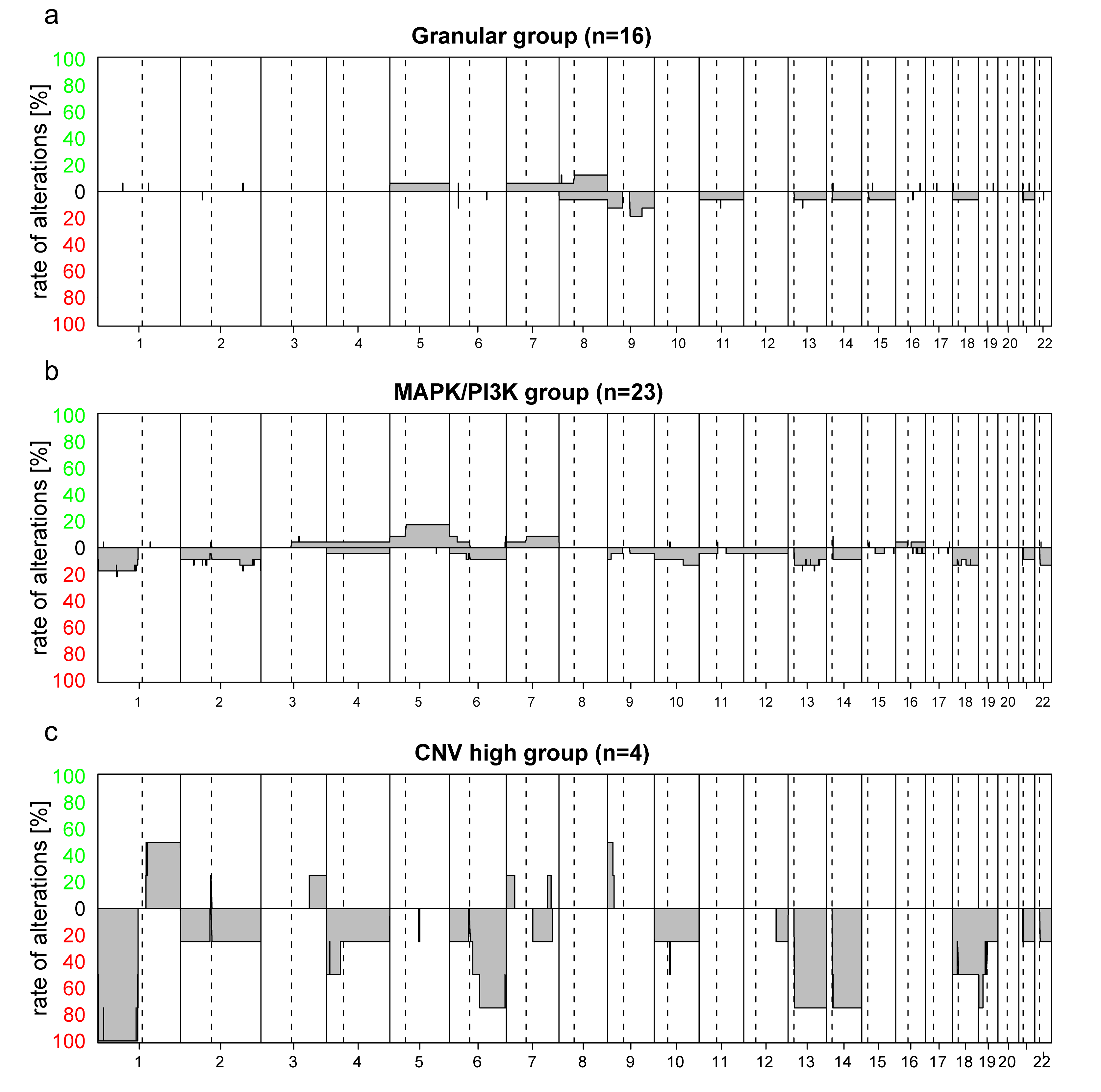

Supplement: Supplementary file 5 — Summary copy number plots for the three posterior pituitary tumor DNA methylation groups. Granular group tumors and MAPK/PI3K group tumors show no highly recurrent cytogenetic alterations. Eleven of sixteen (69%) granular group tumors and 14 of 23 (60%) of MAPK/PI3K group tumors harbored no detectable chromosomal alterations (flat genome) with the remaining cases showing infrequent chromosomal gains or losses. Amplifications or focal deletions were not observed (a, b). In contrast, the small methylation group “CNV high” was characterized by numerous chromosomal gains and losses including loss of chromosome 1p in all four cases(c) (TIF 35106 KB) [file 401_2021_2377_MOESM5_ESM.tif]

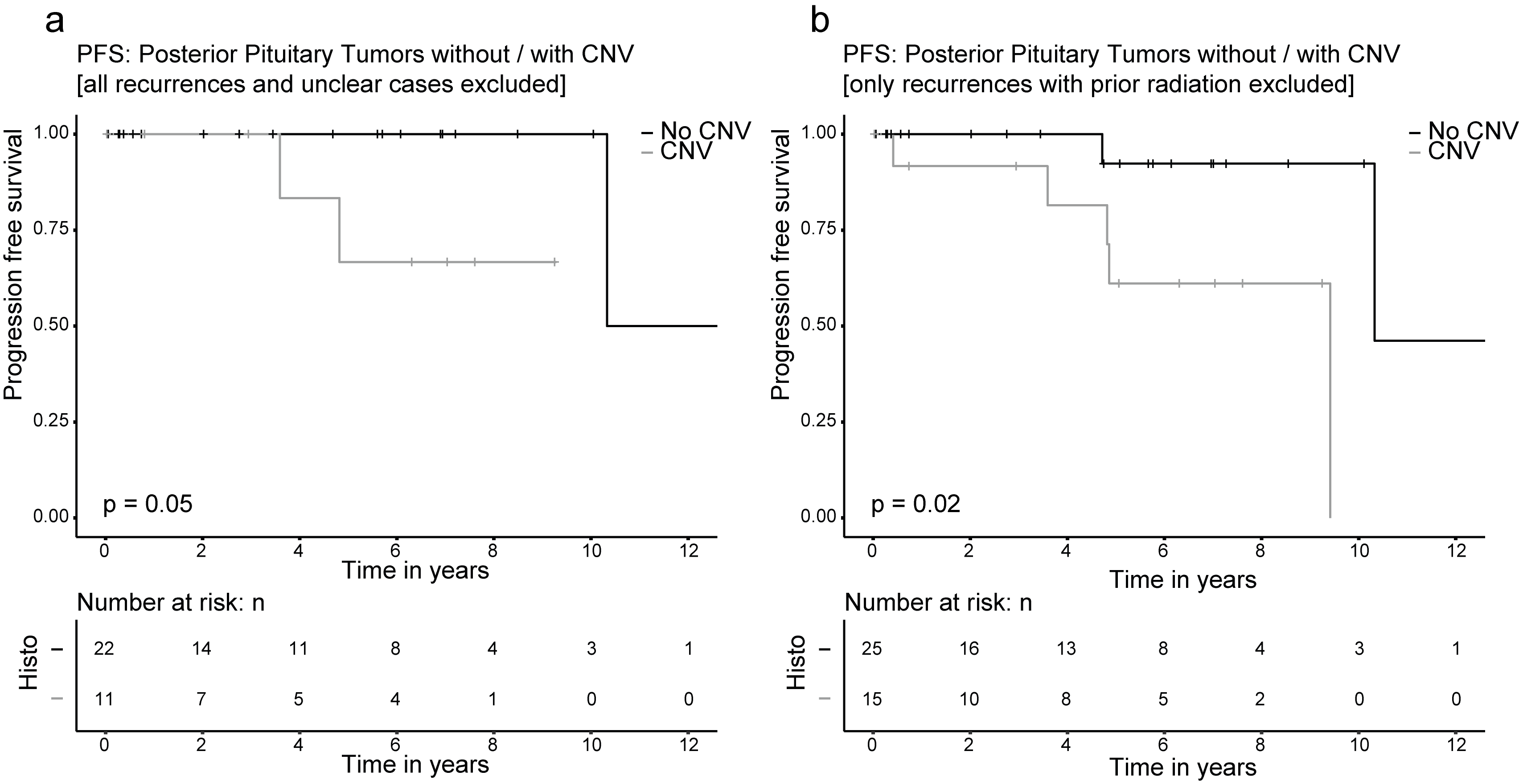

Supplement: Supplementary file 6 — Kaplan-Meier analysis for progression free survival for cases of this series (a) excluding all cases where the initial tumor was not available for analysis or (b) excluding all cases that had a documented radiation therapy prior to sampling. In both subsets the presence of any copy number alterations was still significantly associated with worse outcome (TIF 18465 KB) [file 401_2021_2377_MOESM6_ESM.tif]
